# Role of a Semiotics-Based Curriculum in Empathy Enhancement: A Longitudinal Study in Three Dominican Medical Schools

**DOI:** 10.3389/fpsyg.2017.02018

**Published:** 2017-11-21

**Authors:** Montserrat San-Martín, Roberto Delgado-Bolton, Luis Vivanco

**Affiliations:** ^1^Faculty of Social Sciences of Melilla, University of Granada, Melilla, Spain; ^2^Education Committee Board, University Hospital San Pedro, Logroño, Spain; ^3^Platform of Bioethics and Medical Education, Center for Biomedical Research of La Rioja, Logroño, Spain; ^4^National Centre of Documentation on Bioethics, Logroño, Spain

**Keywords:** communication, curriculum, medical empathy, Dominican Republic, medical semiotics

## Abstract

**Background:** Empathy in the context of patient care is defined as a predominantly cognitive attribute that involves an understanding of the patient’s experiences, concerns, and perspectives, combined with a capacity to communicate this understanding and an intention to help. In medical education, it is recognized that empathy can be improved by interventional approaches. In this sense, a semiotic-based curriculum could be an important didactic tool for improving medical empathy. The main purpose of this study was to determine if in medical schools where a semiotic-based curriculum is offered, the empathetic orientation of medical students improves as a consequence of the acquisition and development of students’ communication skills that are required in clinician–patient encounters.

**Design:** This quasi-experimental study was conducted in three medical schools of the Dominican Republic that offer three different medical curricula: (i) a theoretical and practical semiotic-based curriculum; (ii) a theoretical semiotic-based curriculum; and (iii) a curriculum without semiotic courses. The Jefferson scale of empathy was administered in two different moments to students enrolled in pre-clinical cycles of those institutions. Data was subjected to comparative statistical analysis and logistic regression analysis.

**Results:** The study included 165 students (55 male and 110 female). Comparison analysis showed statistically significant differences in the development of empathy among groups (*p* < 0.001). Logistic regression confirmed that gender, age, and a semiotic-based curriculum contributed toward the enhancement of empathy.

**Conclusion:** These findings demonstrate the importance of medical semiotics as a didactic teaching method for improving beginners’ empathetic orientation in patients’ care.

## Introduction

Professionalism refers to the set of skills and values that, in the case of medicine, characterize the essence of humanism in professional work. This concept arises as an articulated body made up of professional traits and skills that constitute physicians’ professional work regardless of the geographical, social, or cultural settings where it is carried out (Vivanco and Delgado-Bolton, 2015). Medical educators currently are encouraged to make every effort to foster professionalism in medicine by offering programs at the undergraduate, postgraduate and continuing education levels. However, there is still no clear consensus regarding the number and nature of personal qualities required for it. Interpersonal skills as compassionate care and empathy have frequently been mentioned as key components of medical professionalism (Veloski and Hojat, 2006; DeAngelis, 2015).

Why is professionalism relevant in healthcare? A plausible explanation is associated with the importance that the physicians’ understanding means for their patients’ physical, mental, and social needs. Consequently, when clinicians establish empathic relationships with their patients, those basic human needs are fulfilled (Hojat et al., 2003; Soler-Gonzalez et al., 2017). Communication of this understanding is indeed a behavioral aspect of this empathic engagement. Moreover, the central curative aspect of clinician–patient relationships rest not only on the clinicians’ ability to understand their patients but also on their ability to communicate this understanding back to their patient.

Unfortunately, the lack of attention to those skills in some medical programs is partially the result of overreliance on computer-based diagnostic and therapeutic technology. In the biotechnologically advanced atmosphere of patient care, what computers produce seems to receive more attention from some practitioners who trust the machines more than their skills in detecting clinical signs or their patients’ symptoms. In this regard, a recent study demonstrated how patients’ perceptions of the physicians’ empathy predict their health outcomes (Mercer et al., 2016).

### Empathy in Patient Care

According to Hojat (2016, p. 74) empathy in medical settings is “a predominantly cognitive (rather than an affective or emotional) attribute that involves an understanding (rather than feeling) of experiences, concerns and perspectives of the patient, combined with a capacity to communicate this understanding, and an intention to help.” Based on this theoretical framework, researchers and educators in the healthcare professions have attempted to enhance empathy by offering educational programs. Most of them address the broader goal of improving students’ interpersonal skills and understanding which of them implicitly associate an enhancement of the capacity for empathy. It is assumed that this capacity is an essential prerequisite to later demonstrate an empathic behavior (Hojat, 2016, p. 220).

Suchman et al. (1997) proposed an interpersonal model of empathic communication in medical encounters. Emphasis in this educative model is placed on the development of three basic communication skills: “recognizing” patients’ needs (emotions, concerns, and inner experiences); “exploring” them; and “acknowledging” them to generate a positive atmosphere between practitioner and patient during the medical treatment. These three skills correspond, according to Mohammadreza Hojat, author of the Jefferson scale of empathy, to the keywords of “cognition,” “understanding,” and “communicating” that are part of the definition of empathy in the context of patient care (Hojat, 2016). Following this assumption, one of the goals of medical educators is to develop empathic engagement in their students from the early phases of their professional training by flourishing their abilities to recognize “empathic opportunities” when patients express emotions, needs, or concerns. In clinical work, empathic healthcare professionals respond to these “empathic opportunities” offered by their patients by expressing and communicating understanding of their patients’ needs.

### Medical Semiotics

The science of medicine in the treatment of diseases and the art of medicine in the curing of illnesses are not independent entities, they supplement one another (Hojat, 2016). Consequently, symptoms and presentations should belong to the medical signs, independent of the subjective/objective dichotomy and regardless of the negative test results (Malterud, 2000). Human medicine is an enterprise where the object of study is an individual, a human being, not a biological being only. And to understand human beings implies to understand human beings able to understand themselves and able to communicate this understanding. In consequence, this acknowledgment requires practitioners not only to have clinical knowledge, but also understanding abilities, empathic communication, and interpersonal skills, all of which are necessary for establishing an optimal communication with the patient. In this sense, the aim of medical semiotics is to narrow the gap of uncertainty and give a more global understanding of the medical treatment process where symptoms and clinical signs require an interpretation. This first step is crucial in order to optimize physician–patient communication in clinical encounters.

Different authors have recognized the relevance that medical semiotics have in medical affairs (Nessa, 1996; Malterud, 2000; Kugelmann, 2003; Eriksen and Risør, 2014). Due to its role in the interpretation along the entire clinical process, medical semiotics offers to clinicians a wider and more complete scenario to analyze their patients’ health conditions. This integrative scope includes not only biological, but also other factors that are influencing their patients’ health perceptions.

### Medical Education and Interpersonal Skills in the Dominican Republic

By the end of 2015, all the medical schools of the Dominican Republic restructured their academic curricula according to national standards provided by the National Council of Higher Education, Science and Technology (HEST) of the Ministry of HEST. The standards developed by this Ministry establish that a medical program has to reflect the necessary contents to provide professional training that includes the development of professionalism according with international standards. However, the structural design of the medical program is a task that is left to universities themselves. The standards set three main training stages for the medical curriculum: a “pre-medical” phase focused on consolidating and expanding general and multidisciplinary knowledge; a “pre-clinical” phase for the acquisition of biological and biomedical knowledge, as well as for the development of the humanistic competences required for professional practice and dealing with patients; and, finally, a “clinical” phase where training is provided in a clinical or hospital setting. According to this structural framework, the teaching of communication skills, competences in social and community work, or in other human areas of medicine, is not confined to any of the training stages mentioned, and neither are the credits or academic time devoted to such knowledge. In fact, not all the universities arrange their academic years following the same pattern. Finally, and to accommodate their academic offers to a wider market, certain medical schools have chosen to include educational programs designed for foreign realities that could be of professional interest for students, such as the United States, Mexico, or Spain. In the specific case of communication skills, they are mainly taught in theoretical and practical courses of medical semiotics. There is no consensus about when and how those courses should be offered. In some cases they are offered during the “pre-clinical” phase, in others during the “clinical” phase. Finally, there are some schools were those courses are not mandatory.

### The Study

The need to improve the quality of medical education programs has led, in the case of the Dominican Republic, to the development of policies for the assessment and accreditation of the existing academic programs. This has also been reflected in the development of national processes for the standardization, management and assessment of universities. However, the broad flexibility of the criteria applied, and the limited demand from students for a wide and very competitive range of offers due to the high number of private universities, has encouraged many of these institutions to develop creative and innovative initiatives that might differentiate them from other already existing teaching models. Against this background, humanistic areas focused on interpersonal skill development, communication skills, medical ethics, bioethics or medical humanities are especially vulnerable to change, being of particular importance in the context of a reality that is sensitive to the need to promote the training of professionals who are committed with patients and with society’s main healthcare requirements.

The aim of this study was to compare two different pedagogical approaches in the field of interpersonal skill development according to the educative outcomes that they have in the enhancement of medical empathy. Based on the idea that medical empathy is a predominantly cognitive attribute, more than emotional, the following hypothesis was tested: in medical schools where a semiotic-based curriculum is offered, the empathetic orientation of medical students increases as a consequence of the acquisition and improvement of students’ communication skills that are required in clinician–patient encounters.

## Materials and Methods

### Design

The study was based in a quasi-experimental design. It was performed in three medical schools of the 10 existing at that time in the entire territory of the Dominican Republic, between 2014 and 2015. All participating institutions were private.

Participants were students enrolled in the last 4-month academic period of the “pre-clinical” phase in the participating institutions. Two medical schools, where medical semiotics was included in their curricula, were elected as “study groups”; while the third one, without semiotic-based curriculum, was elected as a “control group.” None of the three groups had received previous courses on semiotics or related areas, or had had clinical training experience. Students who were enrolled in extra-academic activities that could influence the enhancement of their empathetic orientation during the same period (i.e., social work programs, volunteering services) were excluded of this study. A brief description of the medical curriculum of each of the three participant institutions during the 4-month academic period studied is presented as follow:

(i) School “A”: The entire period has an academic load of 22 credits. These credits are distributed in: a core semiotics program (10 credits); a course of bioethics with certain contents related to medical semiotics (2 credits); and three courses in areas not related to medical semiotics (10 credits). The core semiotics program is composed of two theoretical courses on medical and surgical semiotics (6 credits), and two practical laboratories on medical and surgical semiotics (4 credits).
(ii) School “B”: The entire period has an academic load of 25 credits. These credits are distributed in a course of medical semiotics (4 credits); a course of medical psychology (3 credits) and a course of preventive medicine and primary care (4 credits), in both cases with some contents related to medical semiotics; and five other courses (14 credits) in technical areas not related to medical semiotics.
(iii) School “C”: In this school a course of medical semiotics is offered only in the “clinical” phase of its academic program. In place of semiotics, the 20 credits of this academic period are distributed in seven courses with contents not related to medical semiotics (20 credits).

Questionnaires were administered by an external researcher at the beginning and at the end of the 4-month academic period during which the study was performed. Both questionnaires were administered on paper to be handed back in closed envelopes. Students’ participation was voluntary and anonymous. The anonymity of students was maintained through the use of pseudonyms. There was no potential harm to participants, and the anonymity was always maintained.

An Ethical Committee for Clinical Research and the Management/Administration of the three participating institutions approved the study design. Likewise, upon agreement, the names and geographical location of the institutions are not provided.

### Instrument

Empathetic orientation was measured using the students’ version of the Jefferson Scale of Empathy (JSE-S). The JSE-S is a psychometrically sound instrument developed specifically to measure empathy of medical students in the context of patient care (Hojat and Gonnella, 2015). The JSE S-Version includes 20 items answered on a seven-point Likert-type scale (1 = strongly disagree, 7 = strongly agree). The possible range of scores is from 20 to 140, and higher scores indicate a higher empathic orientation. The JSE has three components: “perspective taking” (10 positively worded items), the main component of the JSE and the core ingredient of the empathy and the stepping-stone in empathic engagement; “compassionate care” (eight negatively worded items); and “walking in the patient’s shoes” (two positively worded items). According to one of the authors (Hojat, 2016, p. 106), those components are also supportive of the pillars of empathic engagement in patient care, namely mind’s eye in reference to the ability to imagine, remember, or associate images or scenes (e.g., perspective taking and walking in patient’s shoes), and the third ear in reference to personal intuition and sensitivity (e.g., compassionate care). Satisfactory evidence in support of the psychometric properties of the JSE S-Version has been reported in different languages, including in Spanish. A validated Spanish S-Version of the JSE was used in this study (Alcorta-Garza et al., 2005).

Also, information about age and gender were collected through a complementary form.

### Statistical Analysis

A descriptive analysis of the qualitative variables and the scores obtained on the JSE questionnaires by all the participants was carried out. Cronbach’s alpha was used to calculate the reliability of the questionnaires in their first and second administration.

Normality was analyzed to determine the most suitable type of statistical tests for the comparative analysis of the different groups. Logistic regression was used to explain the risk of a negative development of empathy. This led to the creation of a dichotomous variable, with the values “increase” and “decrease” in the development of empathy over time. The remaining variables, age, gender and curriculum model, were treated as explanatory or independent variables. Nagelkerke’s coefficient of determination and the Hosmer–Lemeshow test were used to measure goodness-of-fit. The odds ratio (OR) of the loss of empathy between the two points in time when the research was conducted was determined using the age, gender, and curriculum model variables as a reference.

All analyses were performed using R statistical software, version 3.1.1 for Windows. Multilevel (Bliese, 2013), nortest (Gross et al., 2015), fmsb (Nakazawa, 2017), and binomTools (Hansen et al., 2011) packages were used for the statistical analyses of the data.

## Results

### Participants

The entire population of medical students from the three cohorts who fitted the inclusion criteria for this study, 165 students, agreed to take part in the study. In the first application, all of them completed and returned the questionnaires. In the second application of questionnaires, 164 students completed and returned their surveys. Thirty-five students were enrolled in the school “A” (14 male, 21 female), 21 students were enrolled in school “B” (8 male, 13 female), and 109 students were enrolled in school “C” (33 male, 76 female). In the entire sample, 110 students were female, and the other 55 were male. The mean age was 21 years old with a range of 18–36 years (*SD* = 3.05).

### Reliability

The JSE showed acceptable reliability, given by Cronbach’s alpha coefficient, with values of 0.74 at the beginning and 0.79 at the end of the study. These values were respectively similar and higher than the original results reported by the authors who validated the Spanish version of the JSE in Mexican medical students (Alcorta-Garza et al., 2005). The complete description of the scores of the three components of the JSE, for the whole sample and for groups both at the beginning and at the end of the study is shown in **Table 1**.

**Table 1:** Comparison of the JSE-S scores, at the beginning and at the end of the 4-month academic period of “pre-clinical” phase program, of 165 Dominican medical students.

Medical empathy by groups	PR	Pre-test		Post-test		Significance
		AR	M (*SD*)	AR	M (*SD*)	
*Entire sample* (*n* = 165)					
Perspective taking	10–70	23–70	59 (8)	40–70	61 (6)	^∗∗∗^
Compassionate care	8–56	16–46	41 (9)	18–56	40 (9)	
Walking in the patient’s shoes	2–14	2–14	10 (9)	2–14	9 (3)	^∗∗∗^
*School “A”* (*n* = 35)					
Perspective taking	10–70	38–70	58 (9)	54–70	64 (5)	^∗∗∗^
Compassionate care	8–56	22–55	41 (8)	40–56	50 (4)	^∗∗∗^
Walking in the patient’s shoes	2–14	5–14	10 (4)	4–14	11 (3)	^∗^
*School “B”* (*n* = 21)					
Perspective taking	10–70	40–70	60 (8)	47–70	63 (6)	^∗∗^
Compassionate care	8–56	16–55	41 (9)	34–55	45 (6)	^∗∗∗^
Walking in the patient’s shoes	2–14	2–14	11 (3)	4–14	11 (3)	
*School “C”* (*n* = 109)					
Perspective taking	10–70	23–70	59 (8)	40–70	60 (6)	^∗∗∗^
Compassionate care	8–56	16–56	40 (9)	18–54	35 (8)	^∗∗∗^
Walking in the patient’s shoes	2–14	2–14	10 (3)	2–14	8 (3)	^∗∗∗^

*JSE-S, Jefferson Scale of Empathy S-Version; *n*, sample size; PR, possible range; AR, actual range; M, mean; *SD*, standard deviation; ^∗^*p* < 0.05; ^∗∗^*p <* 0.01; ^∗∗∗^*p <* 0.001.*

### Group Comparison

Since the normality test allowed for the assumption of a normal pattern of distribution of the global JSE scores both at the beginning and at the end of the study, comparisons between groups were conducted using a *t*-test of the mean scores obtained on the JSE. For the analysis of JSE components, since the normality test did not allow for the similar assumption, comparisons were conducted using a Wilcoxon test of the median scores obtained on the JSE.

Comparative analysis of the scores of the JSE showed no significant differences between the group of male and female students, neither at the beginning (*p =* 0.46) nor at the end (*p =* 0.22). No statistically significant differences were found upon comparison of the mean score obtained on the JSE by the whole sample at the beginning and at the end of the study (*p* = 0.64).

For comparison analysis the sample was divided according to age in three groups: (i) students younger than 20 years old, (ii) students between 20 and 24 years old; and (iii) students older than 24 years old. When ANOVA was used to compare the mean JSE scores among these three groups no differences were found at the beginning (*p* = 0.12), although some differences appeared at the end (*p* = 0.008), but only between the first and the second group (*p* = 0.009).

**FIGURE 1:**
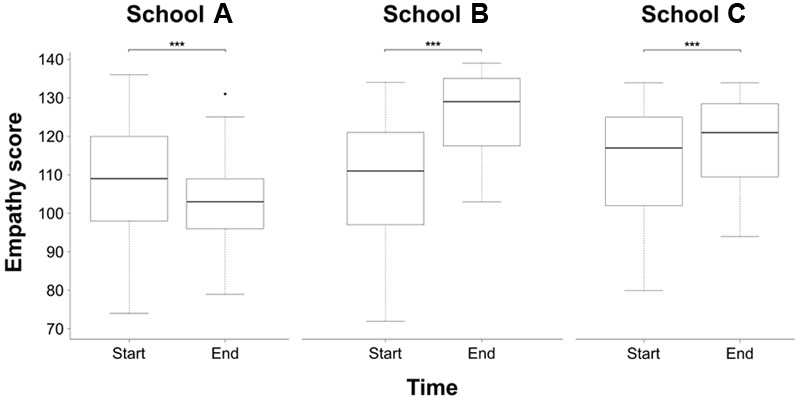
Variation in the score of the JSE-S over time for each of the three schools analyzed. ^∗∗∗^*p* < 0.001.

Statistically significant differences appeared when the comparison of the before and after global scores of the “study” and “control” groups were analyzed (*p* < 0.001), as is shown in **Figure 1**. When ANOVA was used to compare the mean JSE scores among the three groups, it yielded no statistically significant differences at the beginning of the study (*p* = 0.78). However, these differences became statistically significant when the variation in the mean of the JSE was compared over time among the three groups (*p* < 0.001). These differences were observed when variation in the JSE score over time between school “A” and school “C” (*p* < 0.001), and between school “B” and school “C” (*p* < 0.001) were compared. Statistically significant differences were also observed when variation in JSE score between the school “A” and the school “B” were compared (*p* < 0.05). In the entire sample, the analysis of the three measured components of the JSE along time showed a significant increase in the score of the main component “perspective taking” (*p* < 0.001). On the contrary, a significant decrease was observed for the component “walking in patient’s shoes” (*p* < 0.001), whereas no differences were observed for the component “compassionate care” (*p* < 0.11). The analysis by groups showed a significant increase in time in the score of the three components of the JSE in students enrolled in the school “A”; a similar finding was observed in the punctuations of the students enrolled in the school “B” for the components “perspective taking” (*p* = 0.002) and “compassionate care” (*p* < 0.001), but not for the component “walking in patient’s shoes” (*p* = 0.83). Students’ JSE scores from school “C” increased in time only for the component “perspective taking” (*p* < 0.001); on the contrary, a significant decrease was observed in their punctuations for the other two components (*p* < 0.001). A detailed description of these differences is presented in **Table 1**.

**FIGURE 2:**
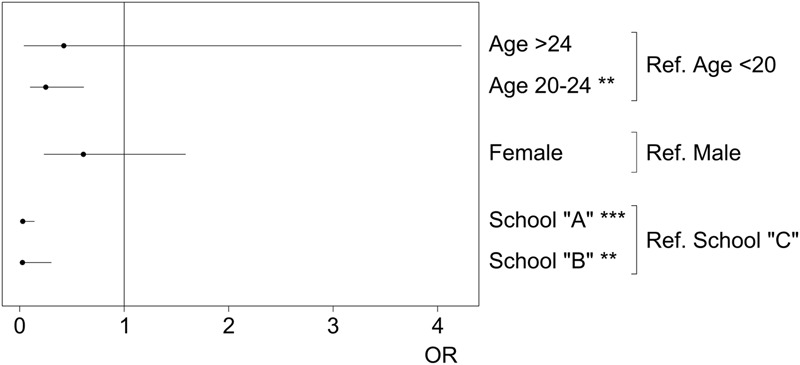
Odd ratios (ORs), 95% CI, of the mean scores obtained on the JSE-S by groups for logistic regression. ^∗∗^*p* < 0.01; ^∗∗∗^*p* < 0.001.

The results of Nagelkerke’s coefficient (*R*^2^ = 0.56) and the Hosmer–Lemeshow test (*p* = 0.94) in logistic regression analysis confirmed an appropriate fit of the data. In the sample studied, the three variables analyzed (age, sex, and curriculum model) contributed to explain variance in the development of empathy. Thus, the risk of loss of measured empathy was lower for students between the ages of 20 and 24 than for those under 20 (OR = 0.25). Meanwhile, the comparison among the groups corresponding to the three curriculum models showed that the risk of loss of measured empathy was considerably lower in students enrolled in medical programs alike to school “A” (OR = 0.03) and “B” (OR = 0.03) than in those enrolled in medical programs alike to school “C.” The complete analysis is presented in **Figure 2**. Based on the results obtained, a *logit* model given by the following equation was produced:

$$\text{P}=1/[1+\text{exp}(b_{0}+b_{1}^{*}{\text{A}}_{2}+b_{2}^{*}{\text{A}}_{3}+b_{3}^{*}\text{F}+b_{4}^{*}{\text{C}}_{\text{A}}+b_{5}^{*}{\text{C}}_{\text{B}})].$$

where, variable “A_2_” represents ages 20 to 24; “A_3_” represents over 24 years of age; “F” corresponds to female; “C_A_” is to study the Medical Degree according to a curriculum model alike to school “A”; “C_B_” is to study the Medical Degree to a curriculum model alike to school “B”; and values “*b*” represent each of the coefficients (*b_*0*_* = -2.4770; *b_*1*_* = 1.3889; *b_*2*_* = 0.8619; *b_*3*_* = 0.4958; *b_*4*_* = 3.5283; *b_*5*_* = 3.6665). This equation allows predicting the probability of loss of measured empathy between the two moments in time for medical students in the Dominican Republic characterized by a certain age, sex and curriculum model variables.

## Discussion

Cronbach alpha values, both at the beginning and at the end of the study, confirm the reliability of the JSE-S when it is applied to medical students of the Dominican Republic, while also reflecting that the participating students gained understanding of the measured concept over time. The latter is possibly a consequence of the learning process and of greater familiarization with the meaning of this professional competence. The value of the mean score obtained on the JSE by the studied sample is slightly above the value reported in the only other study published based on students of medicine of the Dominican Republic, which was done in a different medical school to the three medical schools included in our study (Silva et al., 2014), and similar to the results described by the authors of the validation of the Spanish version of the original scale, carried out with a sample of Mexican medical students (Alcorta-Garza et al., 2005).

The analysis of the whole group and the comparison according to sex at the beginning and end of the study yielded no significant differences. This confirms that the differences among the groups representing the different curricula models are caused by the studied variable rather than by sex differences. The analysis of the initial measured empathy scores shows that the students of all three groups started out with a similar empathy at this point of their professional studies. However, both study groups (school “A” and school “B”) showed an enhancement of empathy over time. Contrary to them, students of the control group (school “C”) showed a clear declination in some necessary components of this attribute over time. In both cases where punctuations of the JSE improved, the greatest increase was observed in students who received theoretical and practical training in communication skills associated to semiotics training programs, which indicates a pedagogical synergy between theoretical and practical knowledge.

Although other interpretations are possible, these findings also bring new evidence in support of the amenability of empathy to change during the course of medical education (Stepien and Baernstein, 2006; Hegazi and Wilson, 2013; Hojat, 2016):

– **Positive change:** The findings observed in both study groups where measured showing an improvement in empathy, confirming the importance of interpersonal skill development as an essential prerequisite to develop empathic behavior. According with the model initially proposed by Suchman et al. (1997), the early development of three basic communication skills, “recognition,” “exploration,” and “communication,” have a positive effect in the early enhancement of empathy in the context of patient’s care. These three elements are explicitly mentioned in the contents of the semiotic programs offered by the medical schools that were part of the study group of this study. These findings also coincide with the suggestion of some authors that didactic teaching methods are effective for improving beginners’ empathic communication skills (Stepien and Baernstein, 2006; Hojat, 2016). On the other hand, advanced techniques, such as role-playing, simulation, and audiovisual methods, are reported to be more useful for advanced training in empathy (Schweller et al., 2014).
– **Negative change:** The decline in measured empathy over time, mainly assessed by the loss in the ability to be sensitive with patient’s concerns that was observed in the control group, is in line with previous international studies where a decline in measured empathy was reported among medical students during the course of their medical education in the absence of a targeted educational program (Hojat et al., 2004, 2009; Neumann et al., 2011; Chen et al., 2012; San-Martín et al., 2016).

The link between empathy and age has been studied with some inconsistent results, especially in undergraduate students. For example, in one study the younger Iranian medical students obtained higher JSE scores than their older peers (Khademalhosseini et al., 2014). On the contrary, older Australian health profession students demonstrated higher JSE scores than younger students (Williams et al., 2014). In another study with Korean medical students no differences were found between empathy and age (Park et al., 2015). Findings on most of these studies are limited because of the restriction of range of age in samples of young students. This study offers new information regarding the role that age plays in the enhancement of empathy at medical schools. The logistic regression analysis evidenced the higher risk of reduction of JSE scores in the younger students’ group, in comparison with the older group. This highlights the role that age plays in the development of empathy. However, more research is needed to completely define the true relationship between empathy and age, not only by using samples with wider range of ages, but also with the inclusion of longitudinal study designs.

Although this study offers empirical evidence in support of the amenability of empathy to change with education, more empirical research is still needed. Studies analyzing the long-term effectiveness of programs designed to enhance empathy with programs focused on developing strategies to maintain the enhancement of empathy along time in undergraduate and postgraduate medical settings are warranted.

### Limitations

Unfortunately, in the Dominican Republic there are important differences in the distribution of the population of students in medical schools. From the 11 universities that now offer schools of medicine in this country, only one is public and the other 10 are private. However, almost half the medical students in the country study in the only existing public school of medicine, all the rest are distributed in private medical schools. Also, the distribution of students in private medical schools is not balanced. For example, the oldest private medical school has the biggest number of students, while the newest medical schools usually have small groups. This study was performed only in private medical schools, where the main differences are due to the different curricula and in their learning methodology. It is possible that in case of a semiotics based curriculum, not only those changes, but also the differences in number of students or group sizes (group of small classes versus group of big classes) may play a role and influence the results. However, the study of this effect may open new areas of research, where the different curricula between the public and private medical schools may also be compared.

## Ethics Statement

This study was carried out in accordance with the recommendations of Comite Ético de Investigación Científica de La Rioja (CEICLAR) with written informed consent from all subjects. All subjects gave written informed consent in accordance with the Declaration of Helsinki. The protocol was approved by the ‘Comite Ético de Investigación Científica de La Rioja (CEICLAR)’ and was approved by the Academic Administration of the Fundacion Universtaria Iberoamericana (FUNIBER) and the three participating Universities in Dominican Republic.

## Author Contributions

LV was in charge of the study’s overall design, coordination with the participating institutions, and drafting of the manuscript. MS-M and LV performed the statistical processing of data. All authors contributed to the presented work, participating during the interpretation process of the results, and approved the final manuscript.

## Conflict of Interest Statement

The authors declare that the research was conducted in the absence of any commercial or financial relationships that could be construed as a potential conflict of interest.
